# The protective role of chicken cathelicidin-1 against *Streptococcus suis* serotype 2 in vitro and in vivo

**DOI:** 10.1186/s13567-023-01199-1

**Published:** 2023-08-21

**Authors:** Yi Lu, Fa Xiang, Liuyi Xu, Hongliang Tian, Qi Tao, Kaixiang Jia, Hang Yin, Chao Ye, Rendong Fang, Lianci Peng

**Affiliations:** 1 Joint International Research Laboratory of Animal Health and Animal Food Safety, College of Veterinary Medicine, Southwest University, Chongqing, 400715 China; 2 Chongqing Key Laboratory of Herbivore Science, Chongqing, 400715 China; 3 WestChina-Frontier PharmaTech Co., Ltd, Chengdu, China

**Keywords:** *Streptococcus suis*, cathelicidin-1, antibacterial activity, anti-inflammatory activity

## Abstract

*Streptococcus suis* serotype 2 (SS2) is an important zoonotic pathogen with the characteristics of high mortality and morbidity, which brings great challenges to prevent and control epidemic disease in the swine industry. Cathelicidins (CATH) are antimicrobial peptides with antimicrobial and immunomodulatory activities. In this study, bactericidal and anti-inflammatory effects of chicken cathelicidin-1 (CATH-1) were investigated in vitro and in vivo against SS2 infection. The results show that CATH-1 exhibited a better bactericidal effect compared to other species’ cathelicidins including chickens (CATH-2, -3, and -B1), mice (CRAMP) and pigs (PMAP-36 and PR-39), which rapidly killed bacteria in 20 min by a time-killing curve assay. Furthermore, CATH-1 destroyed the bacterial morphology and affected bacterial ultrastructure as observed under electron microscopy. Moreover, CATH-1 antibacterial activity in vivo shows that CATH-1 increased survival rate of SS2-infected mice by 60% and significantly reduced the bacterial load in the lungs, liver, spleen, blood, and peritoneal lavage as well as the release of SS2-induced inflammatory cytokines including IL-1α, IL-1β, IL-12, and IL-18. Importantly, CATH-1 did not show severe histopathological changes in mice. Further studies on the mechanism of anti-inflammatory activity show that CATH-1 not only reduced the inflammatory response through direct neutralization, but also by regulating the TLR2/4/NF-κB/ERK pathway. This study provides a scientific basis for the research and development of antimicrobial peptides as new antimicrobial agents.

**Supplementary Information:**

The online version contains supplementary material available at 10.1186/s13567-023-01199-1.

## Introduction

*Streptococcus suis* (*S. suis*) is one of the most important zoonotic bacterial pathogens, which seriously threatens the development of the swine industry, causing huge economic losses in the world [1]. *S. suis* infection causes various diseases, such as septicemia, meningitis, pneumonia, endocarditis, arthritis and toxic shock-like syndrome [2]. Since *S. suis* was first discovered in Denmark in 1968, approximately 1600 *S. suis*-infected cases have been reported in humans [3, 4]. So far, *S. suis* has been classified into 29 serotypes (types 1–19, 21, 23–25, 27–31, and 1/2) based on their capsular antigens, of which serotype 2 of *S. suis* (SS2) isolated from piglets is the most virulent and widely prevalent strain [5, 6]. In 1998 and 2005, two large outbreaks of SS2 occurred in Jiangsu and Sichuan Province in China, causing 14 and 38 deaths, respectively, leading to serious concerns in public health [5, 7]. At present, antibiotics play a leading role in the prevention and treatment of *S. suis* infection [8], but overuse of antibiotics increases the occurrence of antibiotic-resistance. The strains of *S. suis* resistance to different antibiotics such as tetracyclines, macrolides, trimethoprim-sulfonamides, fluoroquinolones, aminoglycosides, β-lactams, and chloramphenicol have been present [9, 10]. Therefore, there is an urgent need to find novel and effective antimicrobial agents to control *S. suis* infection.

Cathelicidins belonging to the family of host defense peptides (HDP) also called antimicrobial peptides are the key components of innate immunity with antimicrobial and immunomodulatory activities [11]. Cathelicidins exist in different species including mammals, birds, fish, reptiles, and amphibians, playing an important role in host defense against invading pathogens [12]. Cathelicidins show a broad spectrum of antimicrobial activity against bacteria, fungi, viruses, and parasites [13]. Unlike most conventional antibiotics that act on limited specific targets, cathelicidins kill bacteria by binding lipopolysaccharides (LPS), acting on cytoplasmic targets (such as DNA and RNA), or binding to gram-positive bacterial cell wall precursor Lipid II and inhibiting cell wall biosynthesis, which unlikely produce drug resistance [14]. Besides their direct antimicrobial activities, cathelicidins also provide protection in the host by exerting immunomodulatory activities. For example, human cathelicidin LL-37 inhibits inflammatory response during *S. aureus* infection by inhibiting the activation of Akt and p38MAPK signal transduction and weakening the release of proinflammatory cytokines [15]. Chicken cathelicidin D-CATH-2 plays an immunomodulatory role in *S. suis* infection by skewing bone marrow-derived dendritic cells towards a more macrophage-like phenotype.

Chicken cathelicidin-1 (CATH-1) is a 26 amino acid cationic linear peptide, consisting of an α-helical segment with a slight kink near the center and linking a flexible unstructured region at the N-terminal [17]. It has been reported that CATH-1 has a broad spectrum of antibacterial activity against *Staphylococcus aureus* (*S. aureus*), *Listeria monocytogenes* (*L. monocytogenes*), *Klebsiella pneumonia* (*K. pneumonia*), *Pseudomonas aeruginosa* (*P. aeruginosa*), *Salmonella Enteritidis* (*S. Enteritidis*), and *Escherichia coli* (*E. coli*) [17–19]. Furthermore, the combination of CATH-1 with erythromycin has good synergistic antibacterial activity against *E. coli* without inducing strong resistance [20]. However, the exact protective mechanism of CATH-1 is rarely studied against bacterial infection in vivo.

In this study, we first investigated the antibacterial effects of CATH-1 against SS2 strains including SC19 and P1/7 in vitro through different assays including a time-killing curve, scanning electron microscopy (SEM) and transmission electron microscopy (TEM). Furthermore, antimicrobial and anti-inflammatory activity of CATH-1 in SS2-infected mice and primary macrophages were investigated. Our study reveals that CATH-1 provides important protection against SS2 in vitro and in vivo by direct killing and anti-inflammatory activity, which provides an important basis for the development of CATH-1 as anti-infectives.

## Materials and methods

### Peptides

All peptides were synthesized by China Peptides (Shanghai, China) using Fmoc-chemistry and purified by reverse phase high-performance liquid chromatography to a purity > 95% (Table 1). Stock solution was prepared in sterile water.

**Table 1:** **Characteristics of peptides used in this study**

Peptide	Amino acid sequence	Length	Charge
CATH-1	RVKRVWPLVIRTVIAGYNLYRAIKKK	26	+ 8
CATH-2	RFGRFLRKIRRFRPKVTITIQGSARF	26	+ 9
CATH-3	RVKRFWPLVPVAINTVAAGINLYKAIRRK	29	+ 7
CATH-B1	PIRNWWIRIWEWLNGIRKRLRQRSPFYVRGHLNVTSTPQP	40	+ 7
CRAMP	GLLRKGGEKIGEKLKKIGQKIKNFFQKLVPQPEQ	34	+ 6
PMAP-36	GRFRRLRKKTRKRLKKIGKVLKWIPPIVGSIPLGCG	36	+ 13
PR-39	RRRPRPPYLPRPRPPPFFPPRLPPRIPPGFPPRFPPRFP	39	+ 10

### Bacterial strains

SS2 strains including SC19 and P1/7 used in this study were kindly provided by Prof. Xiangru Wang (College of veterinary medicine, Huazhong Agricultural University). Tryptic soy broth (TSB; Beijing Land Bridge Technology Co., ltd, China) with 10% fetal bovine serum (FBS; Zhejiang Tianhang Biotechnology Co., ltd, China) were used as bacterial growth media for antimicrobial activity assays and tryptic soy agar (TSA; Beijing Land Bridge Technology Co., ltd, China) plates containing 10% FBS were used for the colony counting assay. Bacteria were cultured at 37 °C for 18 h in TSB or TSA agar plates.

### Mice

Wild-type (WT) C57BL/6 mice were purchased from the Chongqing Academy of Chinese Material Medical (Chongqing, China). TLR2^−/−^ and TLR4^−/−^ mice were kindly provided by Dr Feng Shao from the National Institute of Biological Sciences (Beijing, China). These knockout mice were on a C57BL/6 background and maintained in Specific Pathogen Free (SPF) conditions for use at 8–10 weeks of age. This study was approved by the Institutional Animal Care and Use Committee (IACUC) of Southwest University, Chongqing, China (IACUC-20221022-02).

### Cells

Murine erythrocytes were obtained by collecting blood from the orbital vein. For peritoneal macrophage collection, mice were intraperitoneally injected with 2–3 mL 4% thioglycolate medium (Eiken, Tokyo, Japan). After 3–4 days, mice peritoneal exudate cells were collected by peritoneal lavage and suspended in RPMI1640 + 10% FBS or Opti-MEM (Gibco, USA). Then, cells were seeded at 2 × 10^5^ cells/well in 48-well plates or 1 × 10^6^ cells/well in 12-well plates and maintained at a humidified 37 °C incubator with 5% CO_2_. After 2 h incubation, the nonadherent cells were removed and the adherent cells were used for assays as described below.

PK-15 cells were maintained at 37 °C in 5% CO_2_ and cultured in Dulbecco modified Eagle medium (DMEM; Gibco, CA, USA) supplemented with 10% FBS and antibiotics (100 U penicillin/mL and 100 μg streptomycin/mL). Cells were seeded at 1 × 10^5^ cells/well in 48-well plates and cultured overnight before being used for the assays described below.

### Antimicrobial activity assay

Antimicrobial activity of cathelicidins was performed using a broth microdilution assay. Briefly, bacteria were maintained in TSB medium with 10% FBS at 37 °C and grown to mid-logarithmic growth phase before being tested. Then, 50 μL of peptides (0 to 80 μM) in triplicate were mixed with an equal volume of bacterial suspension (2 × 10^6^ CFU/mL) in 96-well plates and incubated for 18 h at 37 ℃. The minimum inhibitory concentration (MIC) was defined as the lowest concentration of ones at which there was no visible bacteria growth. The minimum bactericidal concentration (MBC) was determined by colony counting. Fifty microliter mixed medium were removed from wells in which visible bacteria growth was not observed and then were plated out on TSA plates. After overnight culture at 37 °C, survival bacteria were counted.

### Time-killing curve assay

Bacterial suspension was prepared as described above. Then, 50 μL CATH-1 at 0.5×, 1×, 2×, and 4× MIC concentrations were incubated with the same volume of bacterial suspension (2 × 10^6^ CFU/mL) in 96-well plates at 37 °C for 0, 5, 10, 20, 30, 60, 120, and 180 min. After incubation at the indicated time, 50 μL mixed medium were removed and diluted tenfold serially in TSB and then plated out on TSA plates. After overnight culture, colony counting was performed.

### Scanning and transmission electron microscopy analysis

Mid-logarithmic phase SC19 cells (1 × 10^8^ CFU/mL) were treated with and without CATH-1 at 2 × MIC concentration in 3 mL culture medium at 37 °C for 2 h. After incubation, bacterial suspension was centrifuged and washed with PBS three times. Then, bacterial pellets were fixed with 1.5 mL 2.5% glutaraldehyde in PBS at 4 °C overnight. Finally, samples were sent to the Lilai biomedicine experiment center (Sichuan, China) for scanning and transmission electron microscopy analysis, respectively.

### Hemolytic activity assay

The whole mice blood was washed three times with PBS and diluted in PBS to obtain 2% red blood cell (RBC) suspension. Subsequently, aliquots of 100 μL RBC suspension were mixed with 100 μL CATH-1 (1.25 to 80 μM) in PBS in polypropylene 96-well microtiter plates and incubated for 1 h at 37 °C. RBC in PBS and in 1% Triton X-100 in PBS were set as the negative and positive control, respectively. Next, the plate was centrifuged for 5 min at 1500 rpm and then 100 μL supernatant was transferred to a new 96-well plate to determine the absorbance at 570 nm. The percentage of hemolysis (%) was calculated using the hemolysis formula (%) = [(OD_570nm_ of sample − OD_570nm_ of negative control)]/[(OD_570nm_ of positive control − OD_570nm_ of negative control)] × 100%.

### Cytotoxicity assay

Mice peritoneal macrophages and PK-15 cells were prepared in 48-well plates as described above. CATH-1 (0 to 40 μM) were added into plates and cultured for 24 h at 37 °C with CO_2_. After 24 h incubation, 150 μL 10% WST-1 reagent was added according to the manufacturer’s protocols. After 20 min incubation, absorbance was measured at 450 nm with a microplate reader (Bio-Rad, Japan) and was corrected for absorbance at 630 nm. Untreated cells were set as a negative control. The percentage of cell viability (%) was calculated using the formula cell viability (%) = (OD_450nm_ of sample)/(OD_450nm_ of control) × 100%.

### Toxicity of CATH-1 in vivo

WT C57BL/6 mice (*n* = 10, each group *n* = 5) were intraperitoneally administered with 10 mg/kg CATH-1 and PBS as the control. Mice behavior and weight were monitored for 7 days. Then, the mice were euthanized using ether inhaled narcosis, and blood was obtained from the orbital vein to evaluate the blood chemistries using an automatic biochemical analyzer (Seamaty, China). Next, their peritoneum was photographed and then lungs, livers, and spleens were fixed in 10% formaldehyde followed by dehydration and then enclosed in paraffin. The tissues were sectioned and stained with hematoxylin and eosin (H&E) prior to histologic analysis.

### The evaluation of antibacterial activity of CATH-1 in vivo

WT C57BL/6 mice (*n* = 30, each group *n* = 10) were intraperitoneally infected with SC19 (2.5 × 10^8^ CFU) and PBS as the blank control. After 2 h infection, mice were treated with 10 mg/kg CATH-1 and PBS as the negative control, respectively. Survival rates of mice were monitored daily for 7 days.

After 8 h infection, mice (*n* = 15, each group *n* = 5) were euthanized using ether inhalated narcosis. Blood, peritoneal lavage fluid, lungs, livers, and spleens were collected to determine the number of bacteria using colony counting assay. Meanwhile, peritoneal lavage fluid was also used to determine the production of cytokines including IL-1α, IL-1β, IL-6, IL-12, TNF-α, and IL-18 using ELISA according to the manufacturer’s protocols. The kits of IL-1α, IL-1β, IL-6, IL-12, and TNF-α were purchased from Invitrogen (CA, USA), and IL-18 was purchased from MultiSciences (Hangzhou, China). For histologic analysis, lungs, livers, and spleens were collected and were fixed in 10% formaldehyde followed by dehydration and then enclosed in paraffin. The tissues were sectioned and stained with H&E prior to histologic analysis to observe histopathological changes.

### Bacterial infection in macrophages

Mice macrophages were prepared in 48-well plates as described above and infected with SC19 at a multiplicity of infection (MOI) of 5 and 10. In co-incubation studies, macrophages were co-incubated CATH-1 (1.25 and 2.5 μM) and SC19 at 37 °C with CO_2_ for 2 and 6 h and then gentamicin (250 μg/mL) was added and incubated for 22 and 18 h. In post-incubation studies, macrophages were infected with SC19 for 2 h and then CATH-1 (1.25 and 2.5 μM) and gentamicin (250 μg/mL) were respectively added and incubated for 2 h. Finally, additional gentamicin (250 μg/mL) was added and continued to incubate for 20 h. In pre-incubation studies, macrophages were incubated with CATH-1 (1.25 and 2.5 μM) for 2 h and then cells were washed with RPMI twice and infected with SC19 for 2 h. Next, gentamicin (250 μg/mL) was added and continued to incubate for 22 h. After incubation, supernatants were collected to determine the production of cytokines including IL-1β, IL-6, and TNF-α using ELISA according to the manufacturer’s protocols.

Adhesion and intracellular colony counting experiments in macrophages were performed as reported previously with some modifications [21]. Briefly, under the co-incubation and pre-incubation conditions, after the treatment of CATH-1 and bacteria, cells were washed and directly lysed in TSB containing 10% FBS and 0.1% Triton X-100 for 10 min. Then, adherent bacteria were performed by colony counting as described above. For intracellular bacteria counting, cells were washed and lysed after gentamicin (250 μg/mL) were added and incubated for 30 min.

### Western blot analysis

Mice macrophages were prepared in 12-well plates as described above and incubated with and without peptides for 2 h at 37 °C. After 2 h incubation, cells were washed with Opti-MEM twice and infected with SC19 at a MOI of 5 for 0, 15, 30, 60, and 120 min to determine different protein expression. Then, the macrophages were lysed by the RIPA buffer with PMSF (Beyotime, Beijing, China) and concentrations were determined using a BCA protein detection kit (Beyotime). The cell lysates were separated by a 10–15% SDS-PAGE gel and subsequently transferred to polyvinylidene difluoride (PVDF) membranes. The membranes were immunoblotted with anti-NF-κB p65 Ab (Bioss, Beijing, China), anti-phospho-NF-κB p65 Ab (Beyotime), anti-ERK1/2 Ab (Bioss), anti-phospho-ERK1/2 Ab (Cell signaling technology, Danvers, MA, USA), anti-TLR2 Ab (Wanlei Life Sciences, Shenyang, China), anti-TLR4 Ab (Santa Cruz Biotechnology, Inc.), and anti-β-actin antibody (Beyotime).

### Statistical analysis

Data are represented as mean ± SEM of three independent experiments (in vitro) for each group (*n* = 3). One-way ANOVA was used to analyze statistical significance among different groups. Statistical significance was shown as *p ≤ 0.05, ** *p* ≤ 0.01, ***p ≤ 0.001, ns = no significance.

## Results

### CATH-1 shows effective SS2killing

In this study, we first explored MIC and MBC of cathelicidins from different animals including chickens (CATH-1, -2, -3, and -B1), mice (CRAMP) and pigs (PMAP-36 and PR-39) against SS2 strains including SC19 and P1/7. As shown in Table 2, CATH-1 had the best anti-SS2 activity with low MIC (5 μM) and MBC (10 μM). MIC and MBC of CATH-2 and -3 were between 10 and 20 μM. However, CATH-B1, CRAMP, PMAP-36, and PR-39 show weak anti-SS2 activity with high MIC (> 40 μΜ) and MBC (> 40 μΜ).

**Table 2:** **Antibacterial activity of characteristics against SC19 and P1/7**

Sample	MIC/MBC (μM) toward bacterial strains			
	SC19		P1/7	
	MIC	MBC	MIC	MBC
CATH-1	5	10	5	10
CATH-2	10	20	10	10
CATH-3	10	20	10	10
CATH-B1	> 40	> 40	> 40	> 40
CRAMP	40	> 40	40	> 40
PMAP-36	40	40	40	> 40
PR-39	> 40	> 40	> 40	> 40

**Figure 1:**
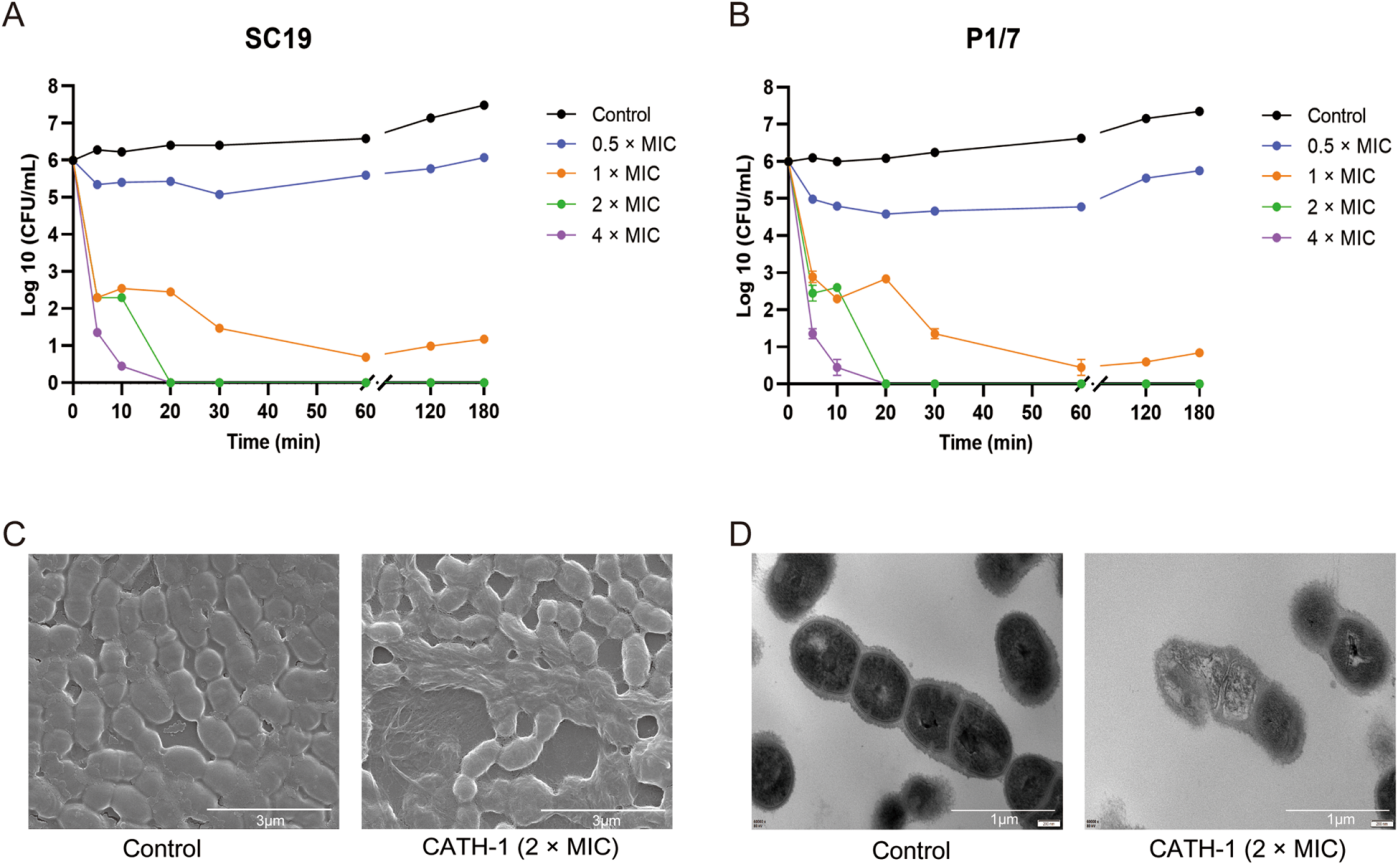
**CATH-1 shows effective killing of SS2.** Time-killing kinetics of CATH-1 against SC19 (**A**) and P1/7 (**B**) are shown. SS2 strain SC19 in mid-logarithmic growth was treated with CATH-1 at 2 × MIC for 2 h. Then, bacterial morphology and ultrastructure were observed by scanning electron microscopy (**C**) and transmission electron microscopy (**D**).

Next, CATH-1 was used to investigate its antibacterial mechanism against SS2 in this study. First, a time-killing assay was performed to examine bactericidal abilities of CATH-1 against SC19 and P1/7. As shown in Figures 1A, B, 0.5 × MIC of CATH-1 failed to show obvious bactericidal activity within 180 min however MIC of CATH-1 had some bactericidal activity. Notably, a high concentration of CATH-1 at 2 × MIC and 4 × MIC effectively killed SC19 and P1/7 within 20 min. Furthermore, the SEM analysis shows that CATH-1-treated SC19 cells exhibited cell membrane damage, cell shrinking, intracellular content leakage and cell lysis (Figure 1C). TEM images of SC19 cells treated with CATH-1 are presented in Figure 1D. Untreated SC19 cells exhibited an intact cellular architecture with a uniform cytoplasmic density, whereas CATH-1-treated cells exhibited ultrastructural damages resulting in breakdown of heterogeneous electron density in the cytoplasm and leakage of the cytoplasmic contents. These results indicate that CATH-1 exerts rapid bactericidal activity by destroying the bacterial morphology of SS2.

### The biocompatible effect of CATH-1 in vitro and in vivo

**Figure 2:**
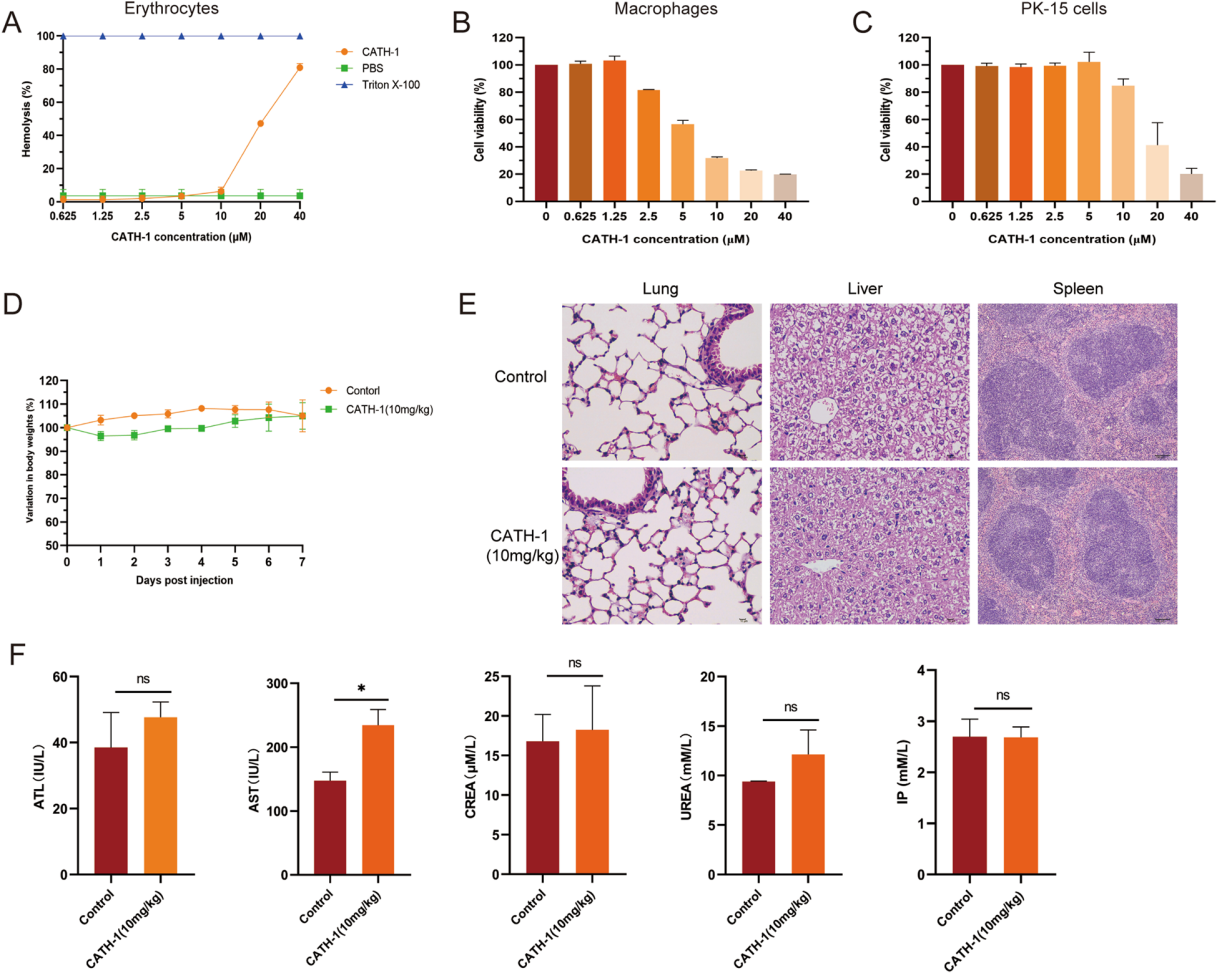
**The biocompatible effect of CATH-1 in vitro and in vivo.** Hemolytic activity of CATH-1 was performed using mice red blood cells (**A**). Cell viability of peritoneal macrophages (**B**) and PK-15 cells (**C**) treated with CATH-1 were tested according to WST-1 assay. Mice (*n* = 10, each group *n* = 5) were intraperitoneally injected with 10 mg/kg CATH-1 and PBS as a negative control. Body weight was monitored daily for 7 days and variation in body weights (%) are shown (**D**). Then, H&E staining was performed to observe histopathological changes in lung, liver, and spleen tissues (**E**) and evaluate the blood chemistries (**F**) at 7 days.

To evaluate the biocompatibility of CATH-1 in vitro, mice erythrocytes and macrophages as well as PK-15 cells were used to determine hemolytic activity and cytotoxicity, respectively. As show in Figure 2A, CATH-1 did not show hemolysis at lower than or equal to 10 μM. Cytotoxicity assay results show that CATH-1 at 5 μM shows toxicity to macrophages (cell viability < 60%, Figure 2B), while CATH-1 at 20 μM shows toxicity to PK-15 cells (cell viability < 60%, Figure 2C).

Next, to further evaluate the biocompatibility of CATH-1 in vivo, CATH-1 (10 mg/kg) was intraperitoneally injected into mice and control group mice were injected with PBS. Body weight of mice were monitored for 7 days, and then the blood chemistries using an automatic biochemical analyzer and the tissues including lung, liver, and spleen were collected for histological H&E analysis. In the blood biochemical indicators, alanine aminotransferase (ALT), aspartate aminotransferase (AST) activity are usually used to assess hepatotoxicity, and creatinine (CREA), urea (UREA) and inorganic phosphate (IP) are correlated with nephrotoxicity. The results show that CATH-1-treated mice slightly decreased by 3.2% bodyweight on days 1 and 2 then started to gain weight again on day 3, and there was no significant weight difference compared with the control group (Figure 2D). Moreover, the results of histological H&E analysis did not show pathological changes in the lungs, liver, and spleen of CATH-1-treated mice (Figure 2E). The pictures of the peritoneum show that there was no obvious pathological change in the peritoneum of the mice treated with CATH-1 compared with the control group (Additional file 1). In addition, as shown in Figure 2F, AST levels in the CATH-1-treated group were higher than the control group, but at the normal level, while there was no significant differences in the ATL, CREA, UREA and IP levels between the control group and CATH-1-treated group, and they were all within the normal levels. These results demonstrate that CATH-1 has negligible side effects and could be developed as anti-infectives without biosafety issues.

### CATH-1 protects mice against SS2 infection

**Figure 3:**
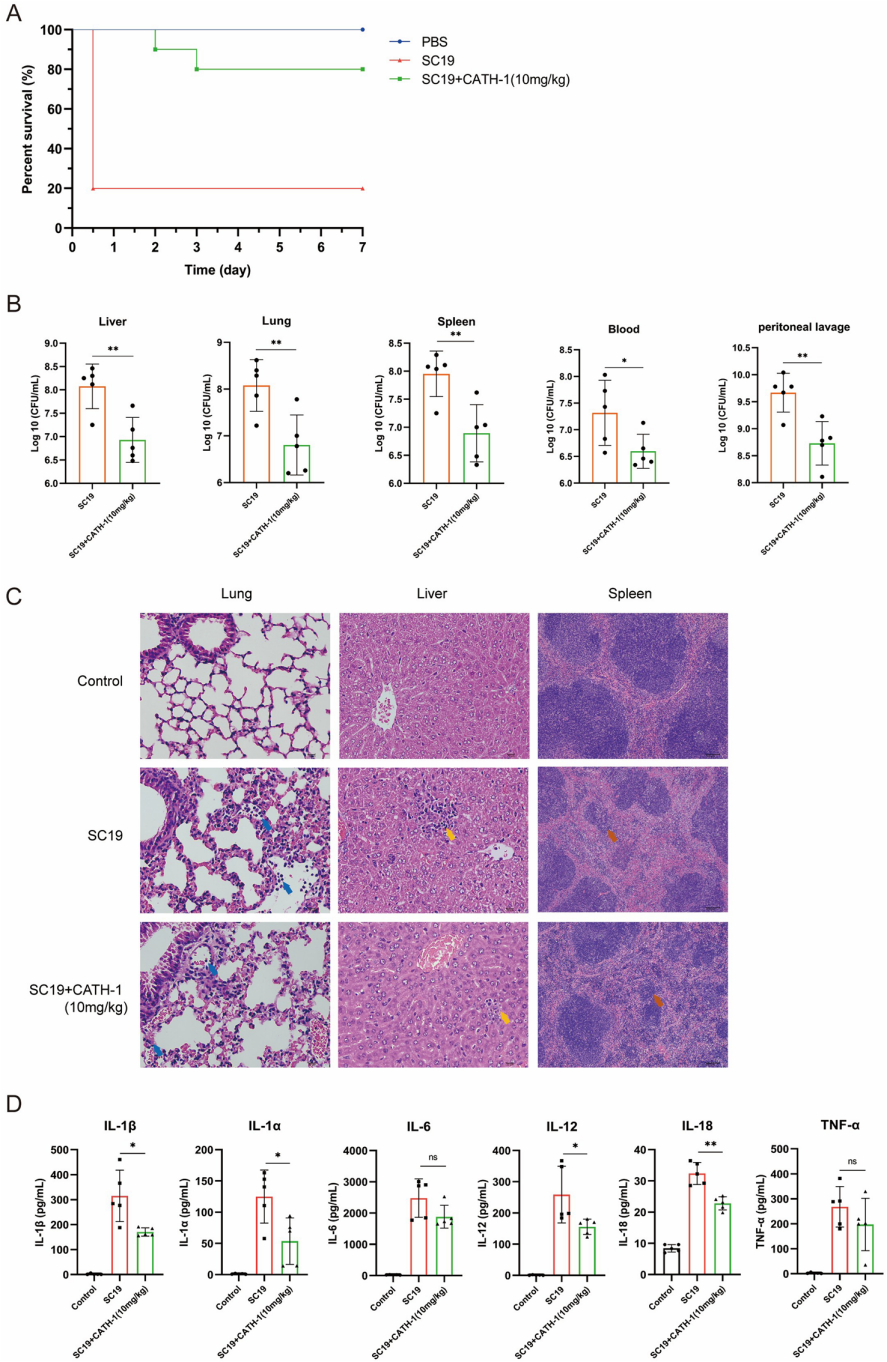
**CATH-1 protects mice against SS2 infection.** Mice (*n* = 30, each group *n* = 10) were intraperitoneally infected with SS2 strain SC19 (2.5 × 10^8^ CFU) and PBS and then intraperitoneally treated with CATH-1 at 2 h post-infection. Mice survival (%) was monitored for 7 days (**A**). After 8 h infection, different samples (*n* = 15, each group *n* = 5) including liver, lung, spleen, blood, and peritoneal lavages were collected and bacterial load was quantified by colony counting assay (**B**). Meanwhile, histopathological changes in liver, lung, and spleen were observed by H&E staining (**C**). In addition, peritoneal lavage was collected at 8 h infection to determine cytokine production including IL-1β, IL-1α, IL-6, IL-12, IL-18, and TNF-α (**D**). Data are represented as means ± SEM of one independent experiment of five mice. *p ≤ 0.05, **p ≤ 0.01, ns, no significance.

To investigate the antibacterial activity of CATH-1 against SS2 infection in vivo, mice were intraperitoneally inoculated with SS2 and then were administrated with CATH-1. Subsequently, mice survival was observed after 7 days infection. As shown in Figure 3A, 80% mice were dead within 12 h after intraperitoneal inoculation with SS2, but mice survival rate was significantly increased by CATH-1 treatment up to 80%, indicating that CATH-1 could protect mice from a lethal SS2 challenge. Next, to determine whether CATH-1 affects bacterial clearance in the host, bacterial load in the liver, lung, spleen, blood, and peritoneal lavage were quantified at 6 h treatment with CATH-1. The results show that bacterial load in the liver, lung, spleen, blood, and peritoneal lavage of CATH-1-treated mice were significantly reduced (Figure 3B), indicating the direct killing role of CATH-1 in vivo.

In addition, H&E staining was performed to observe whether CATH-1 rescues SS2-induced pathological alternation in the liver, lung, and spleen. The results show that SS2 induced inflammatory cell infiltration and increased alveolar interstitials in the lung while CATH-1 treatment decreased SS2-induced inflammatory cell infiltration (Figure 3C, blue arrows). Similarly, CATH-1 treatment alleviated SS2-induced hepatocyte punctate necrosis and disordered cord arrangement (Figure 3C, yellow arrows). In the spleen, SS2-induced the atrophy of nodules and the loss of marginal areas were also alleviated by CATH-1 treatment (Figure 3C, red arrows). These results indicate that CATH-1 attenuated SS2-induced tissue injury.

Inflammatory response is an important strategy to defend invading pathogens, but over production of inflammation induces tissue damage. Therefore, to investigate whether CATH-1 exerts its anti-inflammatory activity in vivo, inflammatory cytokines including IL-1α, IL-1β, IL-6, IL-12, IL-18, and TNF-α in the peritoneal lavage fluid of SS2-infected mice were determined. As shown in Figure 3D, CATH-1 treatment significantly attenuated the production of SS2-induced inflammatory cytokines including IL-1α, IL-1β, IL-12, and IL-18 (except for IL-6 and TNF-α). These results suggest that CATH-1 protect mice from SS2 infection not only through direct killing bacteria but also through its immunomodulatory activity.

### CATH-1 inhibits SS2-induced inflammatory response by direct bactericidal activity

**Figure 4:**
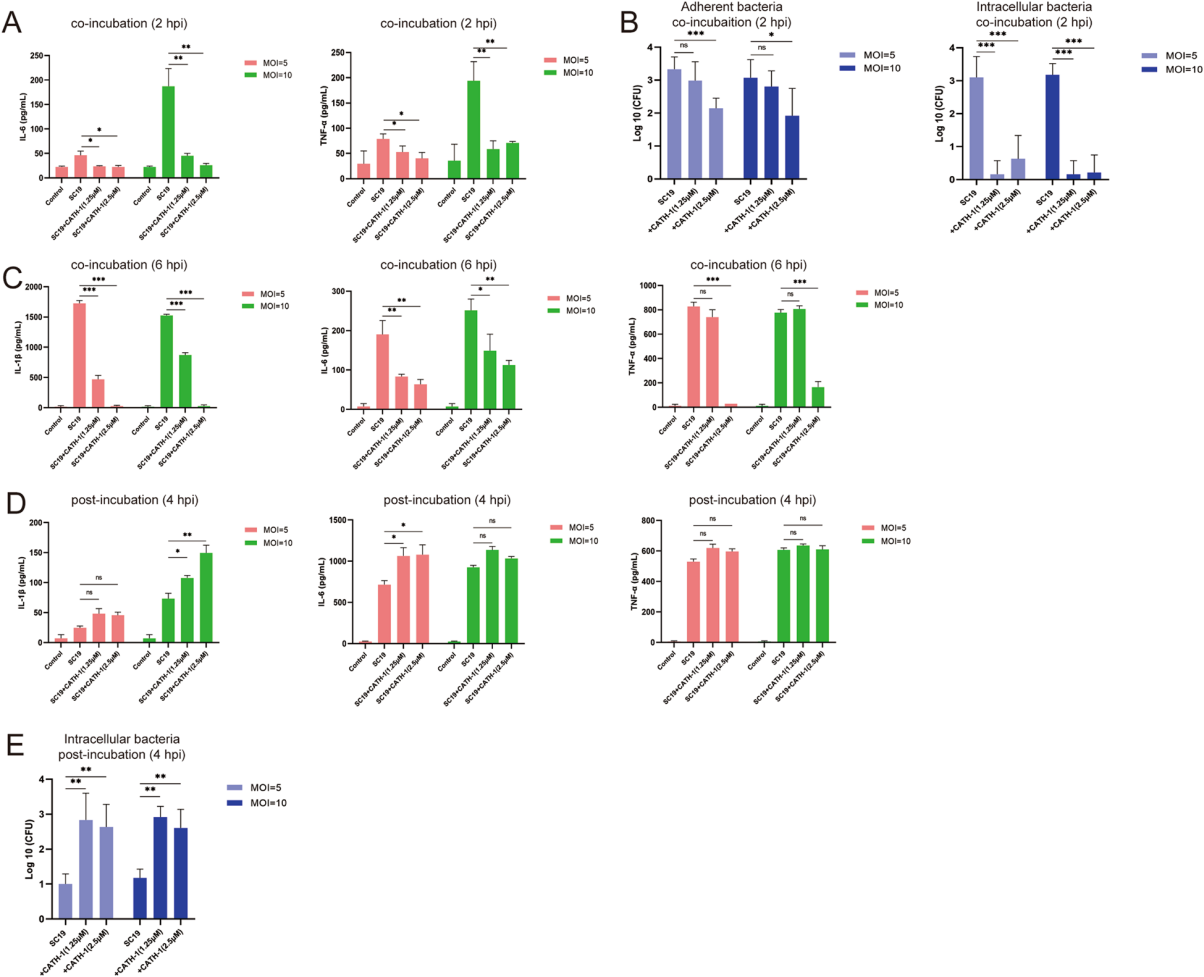
**CATH-1 inhibits SS2-induced inflammatory response by direct bactericidal activity.** Mice peritoneal macrophages were infected with SS2 strain SC19 at a MOI of 5 and 10 in the presence of CATH-1 (1.25 and 2.5 μM) for 2 and 6 h (co-incubation). At 2 and 6 hpi, gentamicin (250 μg/mL) were added and continued to incubate for 22 and 18 h. After 24 h, supernatants were collected to determine cytokine secretion including IL-1β, IL-6, and TNF-α using ELISA (**A** and **C**). Meanwhile, at 2 hpi, partial cells were directly lysed by 0.1% Triton X-100 to quantify adherent bacteria and gentamicin was added to another partial cells for 30 min before lysis to quantify intracellular bacteria (**B**). For post-incubation studies, macrophages were infected with SS2 strain SC19 for 2 h and then CATH-1 (1.25 and 2.5 μM) and gentamicin (250 μg/mL) were added respectively for 2 h. At 4 hpi, gentamicin (250 μg/mL) was added and continued to culture for 20 h. Finally, supernatants were collected to determine cytokine secretion including IL-1β, IL-6, and TNF-α using ELISA (**D**); In addition, at 4 hpi, cells were lysed to quantify intracellular bacteria (**E**). Data are represented as mean ± SEM of three independent experiments of triplicate samples per experiment. * *p* ≤ 0.05, ** *p* ≤ 0.01, *** *p* ≤ 0.001, ns, no significance.

To investigate the exact mechanism by which CATH-1 inhibits SS2-induced inflammatory cytokines in vivo, mice primary macrophages were used to be infected with SS2. The results show that CATH-1 significantly reduced the secretion of IL-6 and TNF-α at 2 h post-infection (hpi, Figure 4A) when CATH-1 was co-incubated with SS2. Under the same infection condition, the number of adherent and intracellular bacteria was significantly decreased by CATH-1 (Figure 4B). Similarly, the secretion of IL-1β, IL-6, and TNF-α was significantly downregulated by CATH-1 at 6 hpi when CATH-1 was co-incubated with SS2 (Figure 4C). However, CATH-1 significantly increased the secretion of IL-1β and IL-6 while it did not change the secretion of TNF-α when CATH-1 was incubated with cells at post-infection (Figure 4D). As with the production of cytokines, CATH-1 significantly increased the number of intracellular bacteria (Figure 4E). These results demonstrate that CATH-1-mediated SS2-induced production of inflammatory cytokines is associated with the number of phagocytosed bacteria, indicating that CATH-1 modulates SS2-induced inflammatory response through direct bactericidal activity.

### CATH-1 inhibits SS2-induced inflammatory response in a TLR2/4-dependent manner

**Figure 5:**
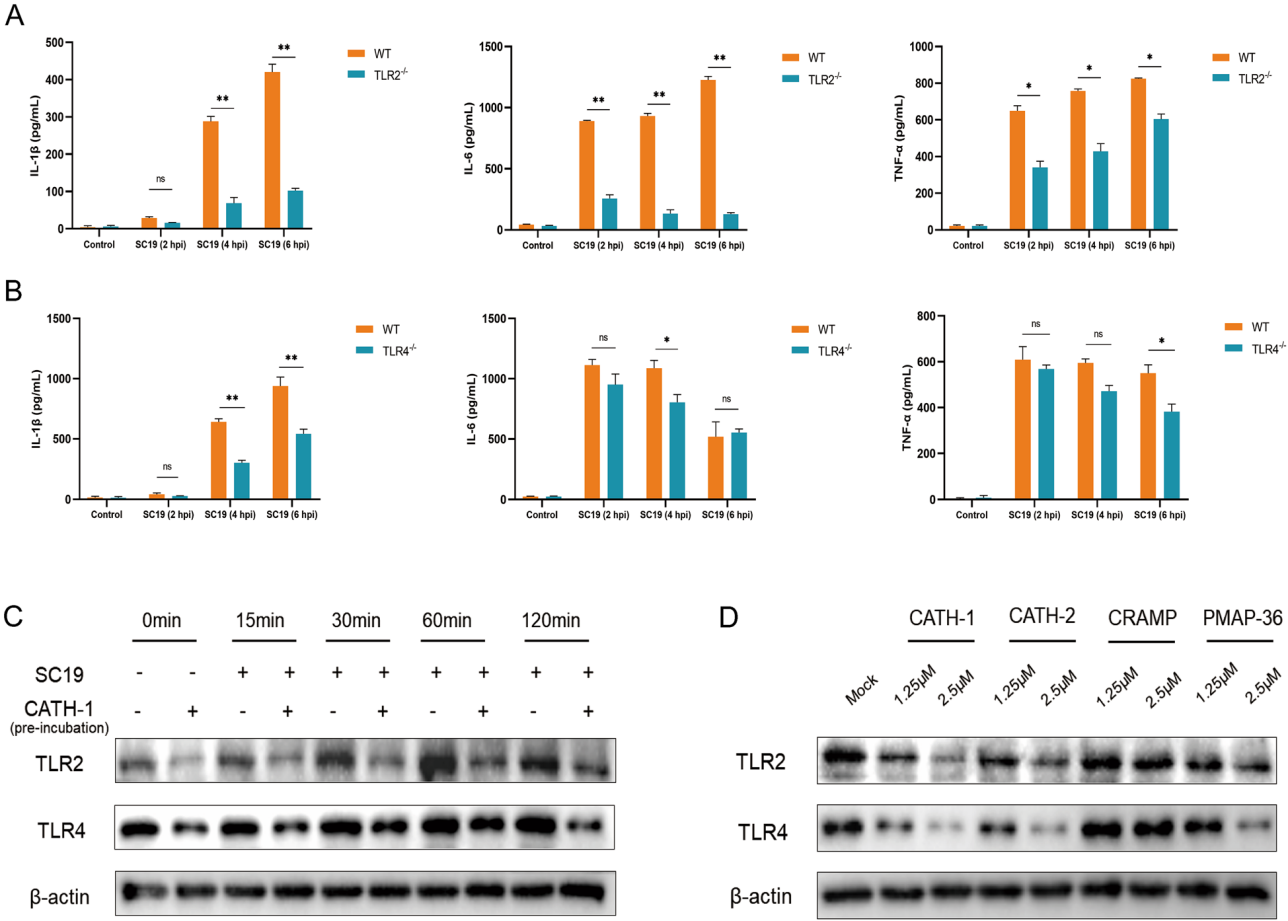
**CATH-1 inhibits SS2-induced inflammatory response via a TLR2/4-dependent manner.** Macrophages from WT, TLR2^−/−^ and TLR4^−/−^ mice were uninfected and infected with SS2 strain SC19 at a MOI of 5 for 2, 4, and 6 h. Then, gentamicin (250 μg/mL) was added and incubated for 22, 20, and 18 h. After 24 h infection, the supernatants were collected to determine the levels of IL-1β, IL-6, and TNF-α in the supernatants (**A** and **B**). WT macrophages were incubated with or without CATH-1, CATH-2, CRAMP, and PAMP-36 for 2 h (pre-incubation). Next, cells were washed with Opti-MEM twice and then infected with SC19 at a MOI of 5 for 0, 15, 30, 60, and 120 min to determine TLR2 and TLR4 protein expression (**C** and **D**). Data are represented as mean ± SEM of three independent experiments of triplicate samples per experiment. * p ≤ 0.05, ** *p* ≤ 0.01, *** p ≤ 0.001, ns, no significance.

To explore the involvement of TLR2/4 in SS2-induced proinflammatory cytokine production, mice primary macrophages from C57BL/6 (WT), TLR2^−/−^ and TLR4^−/−^ were infected with SS2 for 2, 4, and 6 h and then gentamicin (250 μg/mL) was added for an additional 22, 20, and 18 h. Inflammatory cytokine secretion was determined by ELISA. The results show that SS2 induced a high level of secretion of IL-1β, IL-6, and TNF-α in WT mice macrophages, while this cytokine secretion was significantly reduced in TLR2^−/−^ and TLR4^−/−^ macrophages (Figures 5A, B). These results indicate that the production of IL-1β, IL-6, and TNF-α is partially dependent on the activation of TLR2/4 in SS2-infected macrophages. Next, to investigate whether CATH-1-mediated inhibition of SS2-induced cytokine production is dependent on TLR2/4, the expression of TLR2 and TLR4 was detected through pre-incubation CATH-1 with cells 2 h prior to SS2 infection. The results show that CATH-1 significantly abrogated TLR2 and TLR4 expression in SS2-infected macrophages (Figure 5C). Furthermore, the peptides CATH-2 and PMAP-36 downregulated TLR2 and TLR4 expression in a dose-dependent manner while CRAMP did not modulate TLR2 and TLR4 expression (Figure 5D). Taken together, these results suggest that CATH-1 inhibits SS2-induced inflammation via a TLR2/4-dependent manner.

### CATH-1 negatively regulates NF-κB and ERK signaling pathways in SS2-infected macrophages

**Figure 6:**
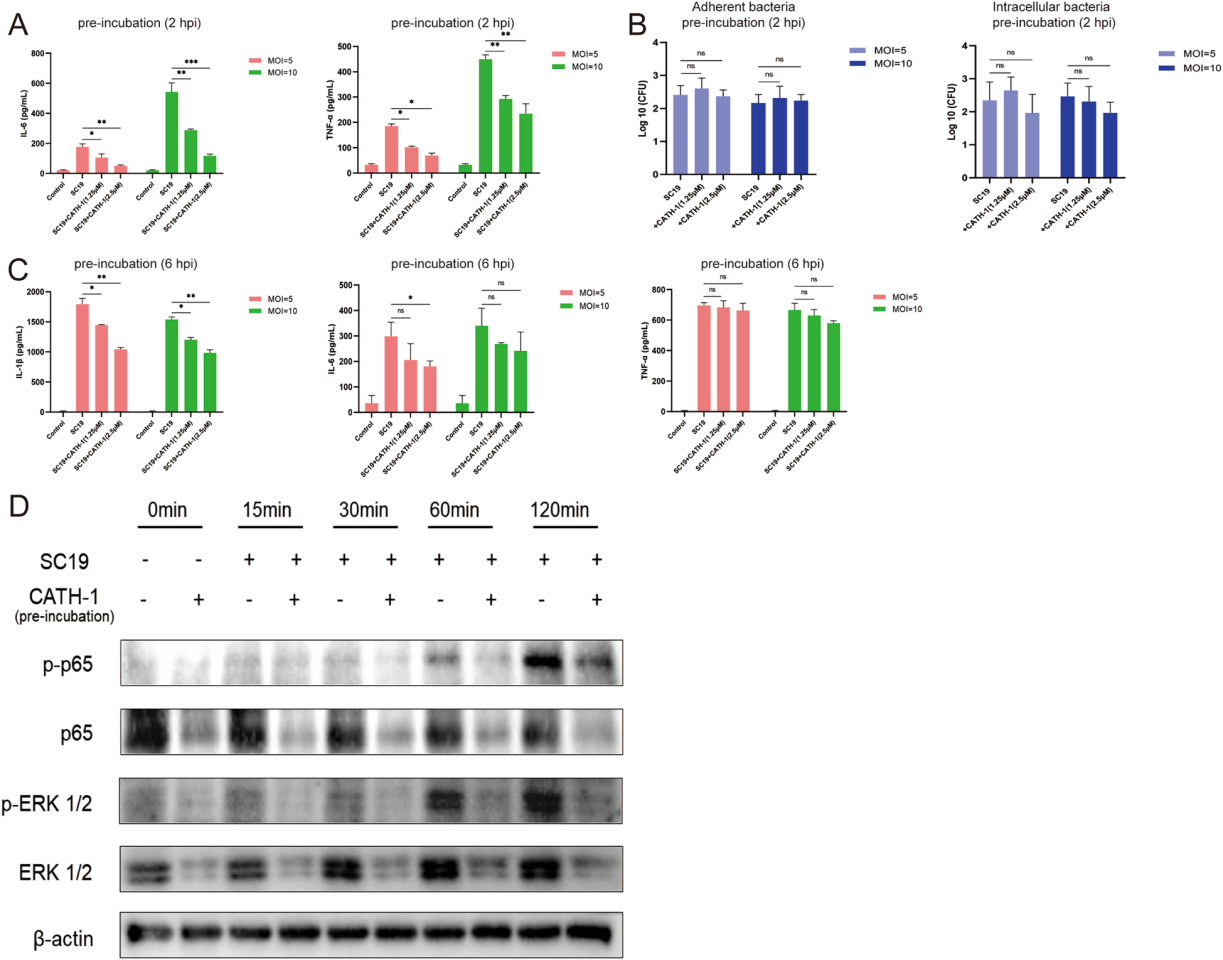
**CATH-1 negatively regulates NF-κB and ERK signaling pathways in SS2-infected macrophages.** Macrophages were incubated with CATH-1 for 2 h (pre-incubation). Then, cells were washed with RPMI twice and then infected with SC19 for 2 and 6 h. At 2 and 6 hpi, gentamicin (250 μg/mL) was added and incubation continued for 22 and 18 h. After 24 h incubation, the supernatants were collected to determine the production levels of IL-1β, IL-6, and TNF-α (**A** and **C**). Meanwhile, at 2 hpi, cells of partial wells were directly lysed by 0.1% Triton X-100 to quantify adherent bacteria and gentamicin was added to another aliquot of cells for 30 min before lysis to quantify intracellular bacteria (**B**). Macrophages were incubated with or without CATH-1 for 2 h (pre-incubation). Subsequently, cells were washed with Opti-MEM twice and infected with SC19 at an MOI of 5 for 0, 15, 30, 60, and 120 min to determine expression of phosphorylation of NF-κB p65 and ERK1/2 (**D**). Data are represented as mean ± SEM of three independent experiments of triplicate samples per experiment. * p ≤ 0.05, ** *p* ≤ 0.01, *** p ≤ 0.001, ns, no significance.

To exclude the effect of CATH-1-induced direct killing bacteria on inflammatory cytokines, CATH-1 was pre-incubated with cells 2 h prior to SS2 infection and then expression of inflammatory cytokines was determined. The results show that CATH-1 still downregulated SS2-induced secretion of IL-1β, IL-6, and TNF-α (Figures 6A, C) while CATH-1 did not affect the number of adherent and intracellular bacteria (Figure 6B), indicating that CATH-1 may exert other actions to inhibit SS2-induced production of IL-1β, IL-6, and TNF-α. Next, we investigated the role of CATH-1 in regulating NF-κB and ERK signalling pathways in SS2-infected macrophages. The results show that the expression of p-p65 and p-ERK1/2 was dramatically up-regulated after 60 and 120 min SS2 infection (Figure 6D) while CATH-1 significantly downregulated their expression. These results suggest CATH-1 inhibits SS2-induced inflammation via NF-κB and ERK signaling pathways.

## Discussion

SS2 is considered as the most virulent serotype of *S. suis*, which has caused huge economic losses in the swine industry and serious public health problems over the past decades [22]. Currently, antibiotics are the main therapies to treat SS2 infection. However, overuse of antibiotics and the slow development of new antimicrobials are the major factors for the emergence of multidrug-resistant strains, and therefore alternative strategies are urgently needed. Cathelicidins belonging to antimicrobial peptides have attracted widespread attention for their strong broad spectrum of antimicrobial activity and their immunomodulatory activity [23]. Therefore, cathelicidins from different animals have been investigated in this study to find one of which has good protection from SS2 infection. It has been reported that chicken CATH-1, -2, and -3 have broad antimicrobial activity [24–26]. Our study shows that chicken CATH-1 exhibited excellent antibacterial activity against SS2 compared to chicken CATH-2 and -3. However, porcine cathelicidins (PMAP-36 and PR-39) show weak antimicrobial activity against SS2, which was consistent with *S. suis* producing dipeptidylpeptidase IV (DPPIV) to inactivate PR-39 [27]. Therefore, it is reasonably speculated that *S. suis* may have a similar escape strategy to inactivate PMAP-36, but the exact mechanism needs to be further studied.

It is well known that antibiotics usually target single structure of bacteria such as cell wall, protein, nucleoids which easily leads to genetic mutations in bacteria and produce drug-resistant strains. Unlike antibiotics, cathelicidins are positively charged and exhibit bactericidal activity by directly disrupting negatively charged bacterial cell membranes, which does not involve specific targets and therefore is less prone to drug resistance [28]. PMAP-36 has been reported to permeabilize the outer membrane of *E. coli* and depolarize the cytoplasmic membrane, thus destroying the membrane integrity and causing intracellular content leakage, which leads to cell death [29]. Schneider et al. found that CATH-2 kills *S. aureus* by penetrating into the cell membrane, resulting in disruption of synthesis of DNA and protein [30]. Similarly, our study shows that CATH-1 completely induced bacterial cell fragmentation, resulting in leakage of bacterial contents and exerting rapid bactericidal effects. Importantly, previous studies have shown that CATH-1 does not induce bacterial resistance against *K. pneumonia*, *S. aureus* and *E. coli* [31]. Notably, our further study shows that the survival rate of SS2-infected mice was significantly improved and bacterial load was also significantly decreased in the liver, lungs, spleen, blood, and peritoneal lavage after CATH-1 treatment, indicating that CATH-1 exerts its protective function in vivo by directly killing bacteria. Similar to other antimicrobial peptides, nisin and plectasin-derived peptide MP1102 treatment via intraperitoneal administration improve the survival rate of *S. suis*-infected mice and bacterial load is also markedly decreased in the liver, lungs, spleen, and blood [32, 33]. Importantly, it has been reported that another chicken cathelicidin (CATH-2) via subcutaneous administration provides immunomodulatory protection such as enhancing the response of macrophages against *S. suis* infection [16]. Compared to subcutaneous and intramuscular administration, intraperitoneal treatment provides more rapid absorption but it is limited to being used in a clinical application. Although our study indicates the rapid and direct antimicrobial activity of CATH-1 in vivo via intraperitoneal administration, whether pretreatment of CATH-1 could prevent bacterial infection via immunomodulatory activity in vivo needs to be further studied.

Excessive inflammation causes tissue damage and accelerates disease progression, which is the serious consequences of *S. suis* infection [34]. Therefore, the inhibition of excessive inflammatory response plays an important role in protecting the host from infection. Jiao et al. found that plectasin-derived peptide NZ2114 clearly reduced SS2-induced production of IL-6 and TNF-α in vivo [35]. Similarly, our study shows that CATH-1 effectively suppressed SS2-induced production of inflammatory cytokines in peritoneal lavage of SS2-infected mice and SS2-infected macrophages. However, the inhibition of CATH-1-mediated inflammatory response was associated with the number of intracellular bacteria and bacterial membrane was disrupted by CATH-1 observed under transmission electron microscopy, indicating the direct interaction of CATH-1 and SS2. Furthermore, it has been reported that CATH-1 neutralizes LTA [17], which is similar to CATH-2 that binds to LPS and LTA and then blocks TLR2 and TLR4 activation, leading to the inhibition of inflammatory response [36]. Importantly, our study shows that SS2 induced inflammatory cytokine production via TLR2 and TLR4 and CATH-1 inhibited TLR2 and TLR4 protein expression, indicating that CATH-1 may bind to TLR2 and TLR4, resulting in the blockage of their activations. Similarly, activation of downstream signaling pathways of TLR such as NF-κB and ERK was also inhibited by CATH-1. Notably, CATH-1 dose-dependently upregulated the secretion of IL-1β when CATH-1 was incubated with cells at post-infection, which is similar to CATH-2 which promotes NLRP3 inflammasome activation, leading to IL-1β secretion [23, 37], suggesting the possibility that CATH-1 serves as a second signal activating inflammasome. These results demonstrate that CATH-1 regulates pro-inflammatory cytokine expression by direct bactericidal effect and modulating TLR2/4-mediated activation of NF-κB and ERK1/2 pathways.

In conclusion, CATH-1 exhibited high bactericidal and anti-inflammatory activity against SS2 in vitro and in vivo. However, the immunomodulatory activity of CATH-1 in vivo should be further studied to develop CATH-1 as a potential therapeutic against microbial infection.

### Supplementary Information

**Additional file 1. CATH-1 treated and untreated mice peritoneal.** Mice were intraperitoneally injected with 10 mg/kg CATH-1 and PBS as negative control. Then, pictures of the peritoneum were observed in the control group (A) and CATH-1-treated group (B) at 7 days.
