# Supplementary figures and images for: The protective role of chicken cathelicidin-1 against Streptococcus suis serotype 2 in vitro and in vivo
Source: Vet Res. 2023 Aug 21;54:65. doi: 10.1186/s13567-023-01199-1 (PMC10463303; doi:10.1186/s13567-023-01199-1)

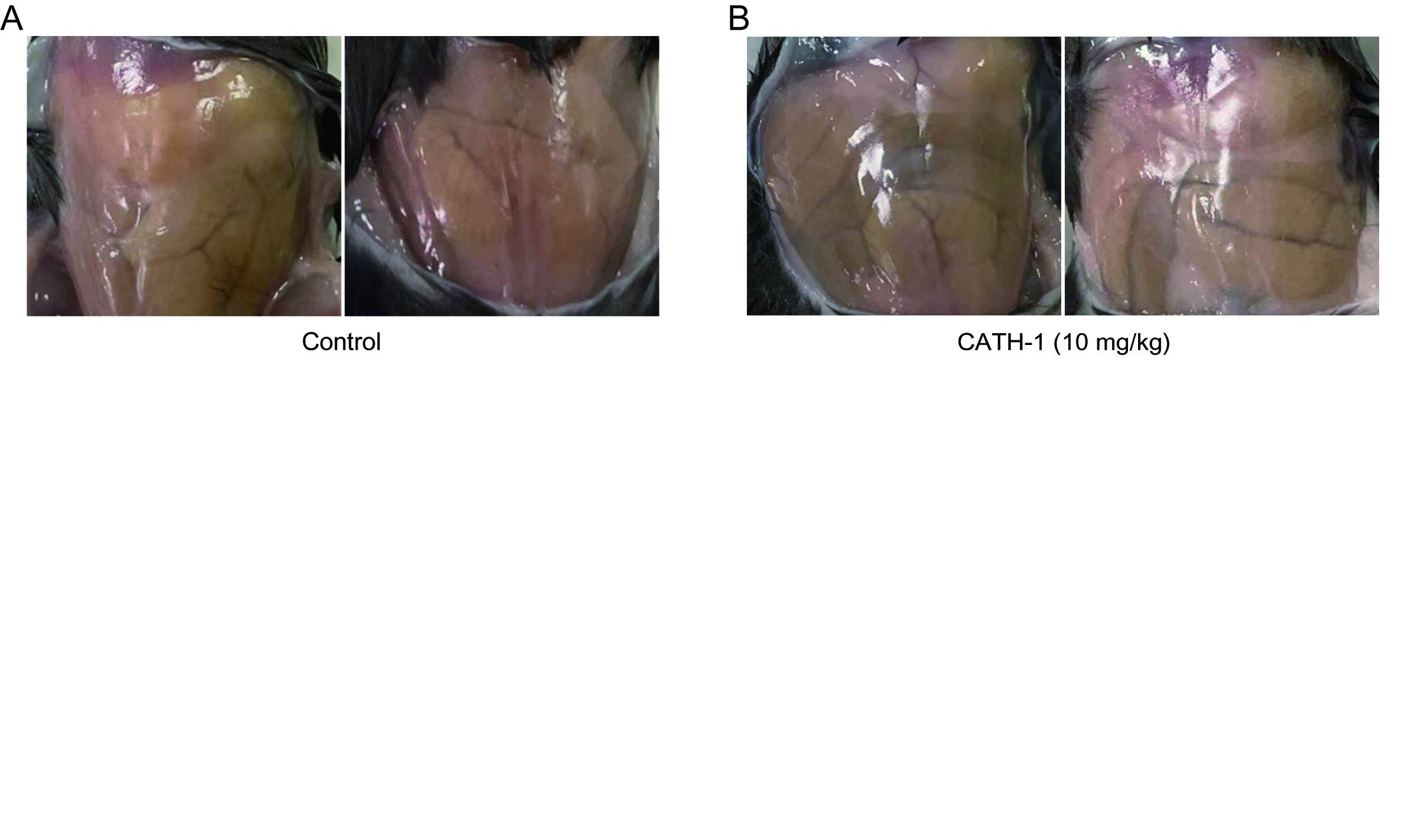

Supplement: Supplementary file 1 — Additional file 1. CATH-1 treated and untreated mice peritoneal. Mice were intraperitoneally injected with 10 mg/kg CATH-1 and PBS as negative control. Then, pictures of the peritoneum were observed in the control group (A) and CATH-1-treated group (B) at 7 days. [file 13567_2023_1199_MOESM1_ESM.tif]
